# Increased nurse workload is associated with bloodstream infections in very low birth weight infants

**DOI:** 10.1038/s41598-019-42685-x

**Published:** 2019-04-19

**Authors:** Erik Küng, Thomas Waldhör, Judith Rittenschober-Böhm, Angelika Berger, Lukas Wisgrill

**Affiliations:** 1 Division of Neonatology, Pediatric Intensive Care & Neuropediatrics, Department of Pediatrics and Adolescent Medicine, Medical University of Vienna, Vienna, Austria; 2 Department of Epidemiology, Center for Public Health, Medical University of Vienna, Vienna, Austria

**Keywords:** Health care economics, Health policy, Neonatal sepsis

## Abstract

Neonatal sepsis is a major cause of morbidity and mortality in very low birth weight infants (VLBWI). Nurse workload considerably affects infection rates in intensive care units. However, data concerning the impact of staff workload on bloodstream infections (BSI) in VLBWI are scarce. The aim of the study was to examine the association between nurse workload and BSI in VLBWI. VLBWI admitted to our neonatal intensive care unit during 2016–2017 were retrospectively analysed. Association between nurse workload, determined by a standardized nursing score, and the BSI occurrence was investigated. A higher nurse workload was significantly associated with higher occurrence of BSI (p = 0.0139) in VLBWI. An assumed workload of 120% or higher, representing the need for additional nurses in our NICU setting, is associated with an elevated risk for BSI in this vulnerable population OR 2.32 (95% CI: 1.42–3.8, p = 0.0005). In conclusion, nurse understaffing is associated with a higher risk for BSI in VLBWI.

## Introduction

Neonatal sepsis and other neonatal infections account for 0.88% of all disability adjusted life years worldwide, demonstrating equivalent percentages compared to motor vehicle road injuries or asthma^1^. Neonatal infections are associated with higher mortality^2^, brain injury^3^, poor neurological development and growth outcomes in early childhood^4^.

Bloodstream infections (BSI) are the most common nosocomial infections in very low birth weight infants (VLBWI)^5^. The median BSI rate for VLBWI was reported to be 10.9 per 1000 patient days^6^ in neonatal intensive care units (NICUs) in the US, 3.3. per 1000 patient days in the German NEO-KISS network^7^ and 2.5 per 1000 patient days in our institution^8^. The vulnerability of VLBWI to infections is caused by multiple factors such as prematurity, immature host defence mechanisms, frequently used antibiotics combined with invasive interventions, and close proximity to other patients.

Transmission of pathogens between patients is mainly caused by hands of health care workers^9–12^. Although this connection is widely known, hand hygiene compliance in NICUs has been reported to be as low as 40%, meaning 60% of recommended hand hygiene steps were not fulfilled^13^. Higher activity levels were shown be associated with lower compliance to hygiene standards^13^. High work load might interfere with every step of nursing performed at the NICU, meaning less effective hand hygiene, suboptimal device insertion and handling as well as suboptimal communication. This chain of correlations leads to the hypothesis, that infection occurrence can be influenced by adjusting the workload of the NICU staff. Facilitating an adequate working level would lead to a lower nurse-patient ratio allowing the staff to focus on the task at hand. Subsequently, the likelihood of accidental spreading of pathogens from one patient to another would be minimized. Therefore, we evaluated and quantified the association between nurse workload and BSI occurrence of VLBWI in our NICU.

## Methods

### Study Design and Setting

This retrospective cohort study was conducted at the Division of Neonatology at the Medical University Vienna/General Hospital Vienna, a tertiary care academic centre, consisting of two separated NICUs (12 and 10 beds, level IV) and two neonatal intermediate care units (NIMCU, 2 × 12 beds). In this study, we analysed data of the NICU (12 beds) directly connected to the obstetric ward exhibiting high admission and transfer rates. All VLBWI admitted to this ward after birth and staying for more than 72 hours between January 2016 and December 2017 were included. High hygiene standards (hygiene training of NICU staff and parents) and standardized antibiotic regimens were maintained during the study period. The study was carried out in accordance with the declaration of Helsinki and approved by the local ethics committee of the Medical University of Vienna (approval number 1859/2018).

### Variables

Demographic and clinical characteristics were routinely collected during the hospital stay using the patient data management system (PDMS) ICCA of Philips Healthcare^14^. Nurse workload was determined using the “TIPPS” (“Tägliches Intensiv Pflege Punkte System”) score, a well-established patient workload classification system^15^. This score is measured as hours spent for patient care per patient day and is based on the “Therapeutic Intervention Scoring System - 28 (TISS)”^16^, modified to include patient contact and administration tasks. The TIPPS-score is calculated using a determined time interval per TISS-point and ward including ICU, NICU or NIMCU. This tool has been used successfully since 1996 by the administration for the determination of staffing levels needed at neonatal and adult intensive care units in the “Viennese Hospital Association (KAV)”. To calculate the work factor used in this study, the work done determined by the TIPPS-score is divided by the hours of staffed nurses in two consecutive 12 hour shifts. In our department, the NICU is staffed with 5 nurses during a night shift and 7 nurses during a day shift. Generally, a work factor of 0.8 corresponds to an overstaffing with no need for one or more staffed nurses and a work factor of 1.2 or higher corresponds to an understaffing and the need for one or more additional nurses. The work factor used to evaluate nurse staffing was calculated once a week, and used for statistical analysis together with the number of infections and the number of patients in the respective week.

BSI was defined according to the NEO-KISS protocol for nosocomial infection surveillance for preterm infants with birthweights <1,500 g^17^. Clinical BSI as well as BSI with coagulase negative staphylococcus (CoNS) were defined as an episode with the following characteristics: occurring after 72 hours of life, empiric antibiotic therapy ≥5d, no apparent infection at another body site and additional two criteria of the following: temperature >38 °C or <36.5 °C, temperature instability, tachycardia, bradycardia, apnoea, hypotension, hyperglycaemia, metabolic acidosis, prolonged recapillarization time or positive blood infection parameter (C-reactive protein >2 mg/dl or IL-6 > 50 pg/ml). Culture-positive BSI was defined as a clinical BSI (see above) with the additional growth of a pathogen in the corresponding blood culture.

BSI occurrence was calculated as number of BSI per number of patients representing a percentage. This calculation differs from the standard calculation of BSI per patient days representing a rate. In this study, we associated the BSI occurrence at time points with the corresponding work factor, therefore we did not use patient days.

### Data analysis

The primary study outcome was the influence of nurse staffing on BSI occurrence in a tertiary NICU with high admission and transfer rates. Continuous variables are presented as mean ± standard deviation (minimum - maximum), count variables are presented as median ± standard deviation (minimum - maximum) and categorical variables as numbers and percentages. Data collected from two consecutive years (2016 and 2017) were analysed. Statistical analysis was performed using the “R” statistic environment^18^ with the “epitools“package^19^. Characteristics of the included neonates and BSI episodes were compared using *χ*^2^*-*test for categorical variables and 2-sample *t*-test for continuous variables. Data is visualized using a Spearman correlation plot, scatterplot with Poisson regression, a time series of occurrence of BSI and work factor as well as a density violin chart combined with a boxplot. Regression analyses was performed using Spearman’s correlation for count data and a Poisson regression using log (number of patients) as an offset variable with a Pearson Chi-square test as goodness-of-fit test. Significance level was set to *p* < 0.05. P-values were not adjusted for multiple testing and have to be interpreted as explorative only.

Data were divided into two groups: adequate work factor (<1.2) and high work factor (≥1.2). This limit would be equivalent to the need for one additional nurse in our setting. To quantify the increased odds for BSI between adequate and high work factor, an odds ratio was calculated using a two by two table. To allow comparison with Leistner *et al*.^20^, a subanalysis using two groups was performed: work load between 90–100% and 100–110%. Between these groups BSI occurrence was compared using a Mann–Whitney-U-test.

## Results

Over two consecutive years, a total of 908 patients were admitted to the NICU. Patient characteristics are shown in Table 1. Mean gestational age of study patients at birth was 28.5 ± 4.5 weeks for 2016 and 28.2 weeks ± 3.8 weeks for 2017. Mean birth weight was 1,148 g ± 731 for 2016 and 1,133 g ± 601 for 2017. Basic characteristics of patients developing a BSI are shown in Table 2. Since there was no significant difference in characteristics of patients with BSI, data were pooled for regression analysis.

**Table 1:** Patient characteristics of patients by year.

Characteristics	2016	2017
Total admissions (patients)	408	500
VLBWI (n)	150	140
ELBWI (n)	106	85
Admissions >72 h (n)	201	178
Gestational age at birth (wks)	28.5 ± 4.5 (23.0–41.3)	28.2 ± 3.8 (23.0–41.4)
Birth weight (g)	1148 ± 731 (440–4,660)	1133 ± 601 (360–3,880)
Length of ward stay (d)	16.6 ± 18.1 (3.0–143.0)	15.4 ± 14.9 (3.0–68.5)

Data shown as number (n) or mean ± standard deviation (minimum − maximum); VLBWI = very low birth weight infant; ELBWI = extremely low birth weight infant; h = hour; wks = week; g = gram.

**Table 2:** Characteristics of patients with bloodstream infections.

Characteristics	2016 (n = 41)	2017 (n = 29)	p-value
Female Gender (n, %)	14 (34.1)	13 (44.8)	0.366
Twin births (n, %)	15 (36.6)	6 (20.7)	0.153
Blood culture positive, (n, %)	21 (52.2)	14 (49.3)	0.121
Death before discharge, (n, %)	5 (12.2)	8 (27.6)	0.103
Gestational age at birth (wks)	25.8 ± 1.8 (23.0–29.4)	25.5 ± 1.9 (23.0–30.0)	0.422
Birth weight (g)	757 ± 188 (440–1,300)	680 ± 182 (470–1,140)	0.095
Patient-days	54.5 ± 19.0 (13–93)	55.1 ± 18.3 (17–81)	0.892
CVC days	41.1 ± 20.9 (6–83)	40.2 ± 15.2 (16–65)	0.804
CVC in place during BSI, (n, %)	37 (90.2)	29 (100)	0.830
PVC days	3.8 ± 5.2 (0–17)	3.7 ± 4.9 (0–15)	0.946
PVC in place during BSI, (n, %)	4 (9.8)	0 (0)	0.136
Days of mechanical ventilation	16.1 ± 16.3 (0–65)	20.6 ± 18.1 (0–53)	0.290
Days of CPAP	31.0 ± 18.2 (0–65)	27.9 ± 16.7 (0–56)	0.467

Data shown as percentage (%) or mean ± standard deviation (minimum – maximum); CVC = central venous catheter; PVC = peripheral venous catheter; BSI = bloodstream infection; CPAP = continuous positive airway pressure.

**Figure 1:**
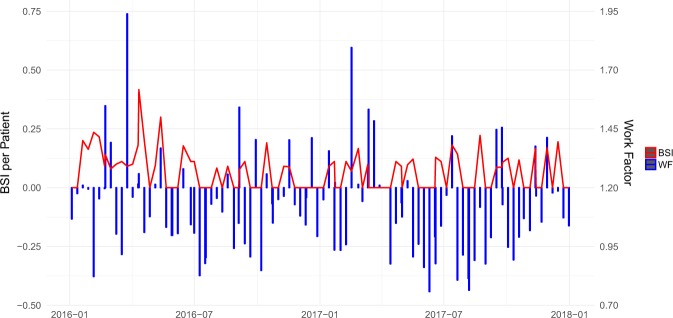
Changes in work factor is associated with changes in blood stream infection rate. Nurse understaffing in the NICU is associated to bloodstream infections (BSI) in very low birth weight infants. Time series of work factor (WF – blue columns) and bloodstream infection (BSI – red line) for the years 2016 and 2017.

**Figure 2:**
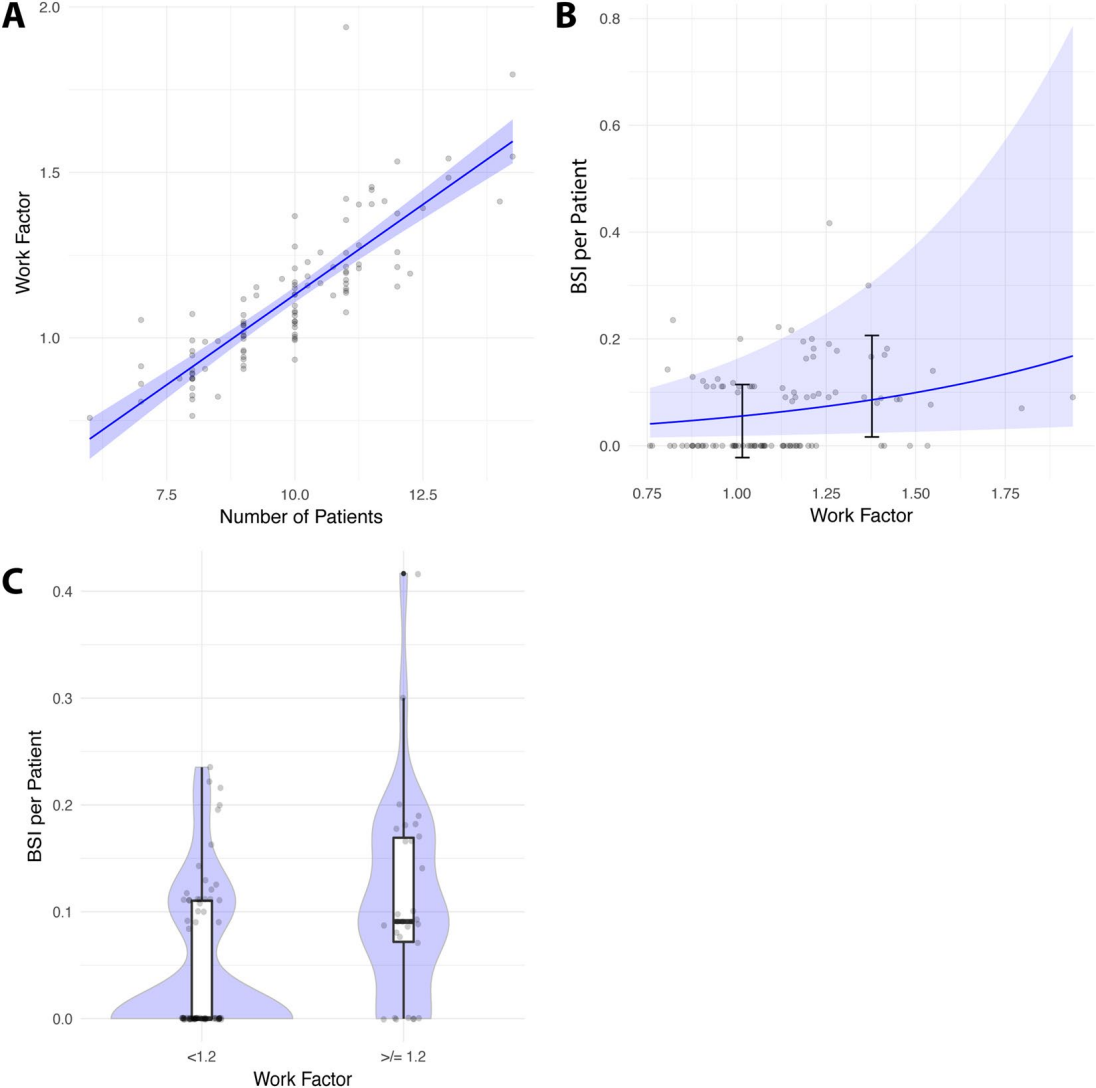
Elevated work factor is associated with higher bloodstream infection rate. Nurse understaffing in the NICU elevates the risk of bloodstream infections (BSI) in very low birth weight infants. (**A**) WF as a function of the number of patients in 2016 and 2017. (**B**) Rate of BSI as a function of WF using a Poisson regression (*p* = 0.0139) with the mean rate of BSI for the five groups of WF. (**C**) Boxplot with violin chart of the rate of BSI for normal (<1.2) and high (≥1.2) WF (p < 0.0010).

Observed work factor and BSI occurrence for the two consecutive years are shown in Fig. 1. The mean work factor over two consecutive years was 1.12 ± 0.21 (CI 95% 0.76–1.94). The observed incidence of BSI was 6.8 per 100 patients. The median number of patients admitted was 10 ± 1.6 (6–14.25) per week. Spearman’s correlation presented a significant (*r*_*s*_ = 0.887, p < 0.0001) strong correlation between the number of patients and work factor as shown in Fig. 2A. This is consistent with the hypothesis of more patients causing more work and thereby a higher work factor. Poisson regression represented a significant (*p* = 0.0139) association between work factor and the BSI occurrence, as shown in Fig. 2B.

Groups with adequate work factor (<1.2) and high work factor (≥1.2) were compared. The distribution of BSI occurrence in each group is shown in Fig. 2C. The Odds-ratio for BSI in the high work factor group was significantly (p = 0.0005) elevated to 2.32 (95% CI 1.42–3.80) compared with the adequate work factor group in a two by two table. The occurrence of BSI did not significantly (p = 0.3984) differ when work load was between 90–100% and 100–110% in a Mann-Whitney-U-test.

## Discussion

Results of the present study indicate a significant association between high nurse workloads and elevated BSI occurrence in VLBWIs. An assumed workload of 120% or higher was associated with a more than twofold increased occurrence of BSIs in this vulnerable patient population highlighting the importance of adequate staff-patient balance in the NICU setting.

There are several studies in adults demonstrating an association between understaffing or high workload in the ICU and an increase in infection rates^21^ as well as adverse outcomes in general^22^. In adults, high nurse workload was shown to be the most important risk factor for healthcare associated infections in the ICU^23^ and significantly correlated with urinary tract infection, surgical site infection^24^, the transmission of methicillin-resistant *Staphylococcus aureus*^25,26^, and increased mortality^27^. Furthermore, understaffing might lead to burnout of the respective nursing staff which is again known to increase infection rates^24^. While this subject is well studied in adults, data on the association between workload and sepsis in premature infants are scarce. This is all the more concerning, since neonatologists are facing particular challenges with regards to infection prevention in the NICU compared to adult ICUs, including patients with an immature immune system and subsequent high susceptibility to infections, often prolonged ICU stays, multiple nurse-patient interactions, a family-centered philosophy of care with a 24 h visiting policy for parents and encouragement of siblings in the ward, as well as mainly open ward designs without single or isolation rooms.

We have shown that an observed workload of 120% is associated with an OR of 2.32 for BSI. In line with these findings, Leistner *et al*. showed that a staffing percentage <95% (equivalent to a realized workload of >105%) resulted in a significant OR of 1.47 for central venous catheter associated bloodstream infections^20^. While the median workload over both years studied in our institution was 108.8% and, thereby, according to Leistner *et al*., associated with a higher risk for infection, we did not find a significant difference in the risk for BSI for a workload of 90–100% and 100–110%. Furthermore, the observed incidence of 6.8 per 100 patients in our cohort was less than half the incidence of BSI (15.2 per 100 patients) reported in the study of Leistner *et al*.^20^, and lower compared to 9.13 per 100 patients reported for our institution in a previous study^8^. Cimiotti *et al*. also reported a correlation between work hours of nurses and rates of BSI in neonates but did not evaluate levels of workload^28^.

Previous studies reported not only infection rates but also mortality rates to be directly related to initial occupancy and nurse-to-infant ratio^29^ as well as understaffing in the NICU setting^30,31^. Understaffing might be accompanied by poor hygiene practices and overcrowding, a combination known to aggravate outbreaks of pathogens in NICUs^32^. In addition, understaffing and overcrowding were shown to cause recurrent outbreaks of *S*. *aureus*^33^. Hensel *et al*. reported on a long-lasting outbreak covering a period of almost 9 months which could only be stopped after substantial recruitment of additional nurses^34^.

All these reports were able to demonstrate an association between infection rates and workload, represented by nurse-patient ratios, patient turn-over and bed occupancy rates in individual studies. By using the same patient classification system as the hospital administration for determining adequate staffing levels, we could calculate a level of work factor or workload for practical usage. Hence, our study is the first to directly associate infection occurrence in the NICU with workload as measured by a standardized calculation tool. The association between workload and infection occurrence can be explained by the decrease of compliance to hygiene measurements^13,35,36^, lack of time to comply with infection control recommendations, job dissatisfaction, job-related burnout, absenteeism, and a high staff turnover^36^.

We observed a wide range of work factor levels with a mean of 1.12. This represents an imbalance of provided nurse working hours and workload and therefore general understaffing in combination with a high fluctuation of workload. These results are consistent with previous studies reporting high fluctuation of workload and high percentages of NICUs (31%) not meeting recommended staffing levels^31,37^.

Although our findings highlight the importance of the nurse work load and associated BSI, our study is limited by its design, grouping and analysed data. While retrospective analysis does not allow conclusions on causality, the number of studies observing similar results, and some of them showing causality in a prospective design, highly suggests the workload to be causative for increased occurrence of infection. The grouping in low (<1.2) and high (≥1.2) work factor was defined by organisational parameters, rather than statistical determination: 20 percentage points is equivalent to the work hours of one nurse. This definition was chosen to provide a practical measurement for nurse staffing in NICUs additionally to the percentage of workload, regardless of staffing practices. The BSI occurrence in one week was compared to the work factor on one day in the same week. While this method does not allow exact determination of correlation, the short time between transmission of pathogens and infection is sufficient for association. Determination of staffing levels is often performed in weekly assessments. Therefore, choosing this method of comparison was necessary to allow implementation in the organisational routine of NICU staffing and present a practicable benchmark for nurse staffing levels in NICUs in general. This analysis of time points does not allow for the calculation of BSI per patient-days (rate). The calculation of occurrence by number of BSI per number of patients (percentage), as used in this study, can lead to bias when patients have different lengths of stay and therefore different risks. By comparing percentages at time points this risk of bias is low. We limited our study to the evaluation of work load and occurrence of BSI. While this allows structured analysis, there is a risk for confounding. From the available data, it is not possible to conclude if the nurse workload or another causative factor (for example physician workload) increases the risk for infection. In regards to the performed studies reporting high nurse workload to be associated with increased risk for infection, workload might be the causative factor, or a risk modulator. Our results are consistent with the results of studies addressing workload and risk of infection in adults. Daud-Gallotti *et al*.^22^ reported nursing workload to be a risk factor for healthcare associated infections in the adult ICU in a prospective cohort study, which makes it plausible to suggest this association in the NICU as well.

Taken together, the increasing number of NICUs unable to meet standards on nurse staffing levels^31^ as well as our results are rather alarming. The evidence that cost-driven downsizing and changes in staffing patterns harm adult patients^21,36^ also seems to be true in neonatal wards. Thus, our results demonstrate that nurse understaffing is associated with a higher risk for BSI in VLBWI, emphasizing the importance of adequate nurse staffing in the neonatal intensive care unit setting.

## Data Availability

The datasets generated during the current study are available from the corresponding author on reasonable request.
